# The Involvement of *Epilobium parviflorum* in Different Human Diseases, with Particular Attention to Its Antioxidant and Anti-Inflammatory Properties and Benefits to Vascular Health

**DOI:** 10.3390/nu17091577

**Published:** 2025-05-03

**Authors:** Klaudia Lewandowska, Michał S. Majewski

**Affiliations:** Department of Pharmacology and Toxicology, Faculty of Medicine, University of Warmia and Mazury in Olsztyn, 10-082 Olsztyn, Poland; michal.majewski@uwm.edu.pl

**Keywords:** aortic rings, *Cuphea carthagenensis*, ellagitannins, Epilobium, hypotension, oenothein B, Onagraceae, polyphenols, systolic blood pressure, vasorelaxation, willow herb

## Abstract

Background/Objectives: Water and alcohol extracts of Epilobium have gained attention due to their high concentration of bioactive compounds and their associated health benefits. This review aimed to evaluate the effects of *Epilobium parviflorum* Schreb. (Onagraceae) preparations on vascular health in light of its medical applications in different human diseases over the last five years. Materials and Methods: A literature search was undertaken of databases such as PubMed/Medline, Scopus, and Google Scholar for original articles published between March 2000 and March 2025. The keywords used were “aortic rings”, “ellagitannins”, “Epilobium”, “hydrolyzable tannins”, “hypotension”, “oenothein B”, “Onagraceae”, “systolic blood pressure”, “vasorelaxation”, and “willow herb”. Results: The *E. parviflorum* Schreb. herb has been used as a remedy in folk medicine and has a variety of therapeutic properties. These include its preventive effects and ability to relieve symptoms in patients with benign prostate hyperplasia, prostatitis, and a variety of cancers. Other properties include effects on kidney and urinary tract diseases, lipid regulation, and skin infections. The herb also has antibacterial properties. *E. parviflorum* contains bioactive compounds such as oenothein B, quercetin-3-O-glucuronide, and myricetin-3-O-rhamnoside. At low doses, these compounds contribute to a reduction in oxidative stress due to their antioxidant and immunostimulatory effects, positively reducing inflammation, which can cause certain conditions. At higher concentrations, Epilobium generates reactive oxygen species that stimulate the body’s defense mechanisms against a variety of cancers. The presence of oenothein B in *E. parviflorum* may influence the production and storage of nitric oxide, which, in turn, promotes vasodilation and regulates blood pressure. Conclusions: Although the potential application of *E. parviflorum* in metabolic disorders has not been extensively studied before, its antioxidant and anti-inflammatory properties are well documented and suggest potential pathways for future research and the therapeutic development of preparations to benefit vascular health.

## 1. Introduction

*Epilobium parviflorum* Schreb., also known as willow herb (UK) or fireweed (US), is a perennial herbaceous plant from the Onagraceae family that grows throughout Europe, Northern Africa, and Western Asia. Tea brewed from the *E. parviflorum* herb offers numerous health benefits, including alleviating disorders of the prostate gland, bladder, and kidney due to the anti-inflammatory, diuretic, and antioxidant activities of *E. parviflorum*. In traditional medicine, an infusion of 1–2 g of *Epilobium* in a cup of water taken orally three to four times daily [1] is prepared mainly to treat patients in the early stages of benign prostate hyperplasia (BPH), urethritis, and micturition disorders [2], as well as in alleviating menstrual disorders [3], rectal bleeding [4] and for the treatment of prostate adenoma [5]. It is also used in treating gastrointestinal and mucous membrane damage and promoting wound healing [4].

Epilobium species are rich in various bioactive compounds such as polyphenols, which are represented by flavonoids (myricetin, quercetin, kaempferol, and catechin), phenolic acids (gallic acid, chlorogenic acid, (Z)-p-coumaric acid, caffeoylquinic acid, p-coumaroylquinic acid, and feruloylquinic acid), and hydrolyzable tannins (ellagitannins) [6]. Other constituents such as lignans, steroids, triterpenoids, fatty acids, and essential oils have also been identified [5,6]. Phytochemical analysis of *E. parviflorum* extracts has shown that oenothein B (ellagitannin) is the predominant compound, ranging from 20% to 35% of the total content in extracts and varies from 2 to 4.5% in the raw plant [6,7].

Oenothein B stands out as a distinct type of hydrolyzable tannin that is characterized by a macrocyclic structure that limits the flexibility of its rotational bonds. This compound is particularly abundant in various medicinal plants such as Epilobium, Oenothera, and Eucalyptus species. Extensive research has demonstrated its diverse biological effects, including antioxidant and anti-inflammatory properties, anticancer activity, immunomodulatory effects, and antibacterial qualities [8].

Phenolic acids are another important contributor to the antioxidant and therapeutic potential of *E. parviflorum,* making it useful for the therapeutic development of preparations benefiting vascular health [9].

## 2. Materials and Methods

A systematic literature search was performed across Scopus, PubMed/MEDLINE, and Google Scholar to identify original research articles published between March 2000 and March 2025. The keywords used were “aortic rings”, “ellagitannins”, “Epilobium”, “hydrolyzable tannins”, “hypotension”, “oenothein B”, “Onagraceae”, “systolic blood pressure”, “vasorelaxation”, and “willow herb”.

## 3. Results

The possible impact of Epilobium on the vasculature, based on its composition and therapeutic properties, has been discussed. The presence of oenothein B in *E. parviflorum* may influence the production and storage of nitric oxide (NO), which, in turn, promotes vasodilation and blood pressure regulation (see Table 1).

### 3.1. Vasodilatory Properties of Oenothein B, the Active Compound of Epilobium

A study conducted by Isla et al. (2024) demonstrated the vasodilatory effects of oenothein B, woodfordin C, and eucalbanin B extracted from *Cuphea carthagenensis* (Jacq.) J. F. Macbr (Lythraceae) [10] (Table 1). *C. carthagenensis* is a popular plant in Brazilian folk medicine that is known for its ability to lower blood pressure and depress activity in the central nervous system. The obtained results showed the pressure-lowering effect of *C. carthagenensis* aqueous extract after one week of oral treatment in normotensive rats. In the same experiment, the rat aortic rings were pre-contracted with noradrenaline and treated with three hydrolyzable tannins—oenothein B, woodfordin C, and eucalbanin B (20–180 µM)—which caused vasorelaxation in a concentration-dependent manner. Hydrolyzable tannins (ellagitannins) have been found to induce in vitro vasorelaxation via endothelial NO synthesis and/or release without impacting Ca^2+^ influx in vascular smooth muscles [10]. According to the authors, the identified in vitro actions of these three compounds are unlikely to account for the hypotensive effect of alcoholic extract in vivo considering the low oral bioavailability of hydrolyzable tannins (ellagitannins). It was found that other compounds and/or mechanisms are involved. Since oenothein B is the main constituent of *E. parviflorum*, it can be concluded that Epilobium herbal extract may also possess hypotensive properties in vivo and regulate NO synthesis and/or release in vitro in a dose-dependent manner. The probable inhibition and/or release of NO involving the *E. parviflorum* mechanism likely depends on the tissue’s oxygenation status, oxidative stress, inflammation, and concentration of reactive oxygen species (ROS), but further studies are required to determine the specifics in more detail.

Nitric oxide is a key signaling molecule playing a role in various biological activities. It regulates vascular tone and blood flow by activating soluble guanylate cyclase (sGC) in vascular smooth muscle, controls mitochondrial oxygen consumption by inhibiting cytochrome c oxidase, and activates immune cells, particularly macrophages, to elicit a protective response. Disturbances in NO production and transport within the vascular area lead to cardiovascular diseases, including hypertension, atherosclerosis, and angiogenesis-related disorders [11].

In another study, Kim et al. [12] described how an ethanol extract of *Oenothera odorata* (Onagraceae) seeds induced vasorelaxation via an endothelium-dependent NO-cGMP signaling cascade through activation of the Akt-eNOS-sGC pathway.

### 3.2. The Antioxidant and Anti-Inflammatory Activity of Epilobium and Its Active Compounds

The antioxidant [1,6,13,14] and anti-inflammatory [8] activities of Epilobium extracts constitute some fundamental biological properties in a plethora of Epilobium species [1]. Myricetin-3-O-rhamnoside (*E. parviflorum*) and quercetin-3-O-glucuronide (*E. angustifolium*) were shown to inhibit cyclooxygenases-1 and -2 (COX-1 and COX-2) [15]. In another study, the methanol–aqueous extract from *E. angustifolium* aerial parts scavenged free radicals, and exerted a distinct impact on α-glucosidase, α-amylase, and lipase. The studied methanol extract also inhibited acetyl- and butyrylcholinesterase and had a prominent anti-tyrosinase effect [6]. Hence, Epilobium extracts may be considered a source of agents for treating disorders related to oxidative stress and inflammation.

The antioxidant and anti-inflammatory effects of *E. parviflorum* and another two plant extracts, *Melilotus officinalis* and *Cardiospermum halicacabum*, showed a beneficial effect in macrophage and microglial cells. Merighi et al. [16] showed that 40% ethanol plant extracts from these herbs reduced NO production in macrophage and microglial cells with a conclusion that these extracts could represent a source of compounds targeting oxidative stress- and inflammation-related disorders. Ethanol extracts from the dried aerial parts of *E. parviflorum* are rich in polyphenols, particularly flavonoids and condensed tannins (see Introduction), and show potential for use in the treatment of disorders associated with oxidative stress and inflammation [7].

In other studies, oenothein B (an ellagitannin-based molecule) was shown to be capable of inhibiting myeloperoxidase, hyaluronidase, and lipoxygenase-5 (LOX-5) [7,17], thereby inhibiting the formation of reactive oxygen species (ROS). Disruption in the body’s antioxidant defense system is linked to the development of diseases such as cardiovascular diseases, inflammation, and cancers. These pathologies occur through an increase in ROS formation [18].

Hiermann et al. [8] noticed that the aqueous but not the methanolic extracts of *E. angustifolii* herbs reduced the release of prostaglandins I2, E2, and D2 (in perfused rabbit ears) approximately five-times more effectively than did similar extracts of *E. parviflori* herbs. Hiermann et al. isolated a minor component, myricetin 3-O-glucuronide, from *E. angustifolium* and identified it as a potent inhibitor of COX-1, COX-2, and 5-LOX activity [8]. Unfortunately, this discovery did not fully explain the inhibition of COX-1 and COX-2 activities by extracts of *E. parviflorum* [1,15]. Ethanol extracts of *E. parviflorum* also exhibit inhibitory effects on both COX-1 and COX-2-catalyzed prostaglandin biosynthesis and exert antioxidant activity [1]. COX-1 functioning is typically associated with maintaining physiological homeostasis and tissue protection, whereas COX-2 is the primary enzyme responsible for prostaglandin production in response to inflammation [19]. However, the high activity of COX enzymes, particularly COX-2 (and in some cases COX-1), can impact the development of arterial hypertension. Both COX isoforms are involved in prostaglandin synthesis, which affects the regulation of blood vessel tension. Prostaglandins can act on vascular receptors, leading to impaired vasoconstriction and/or vasodilation. Additionally, they may influence sodium and water retention in the body, thereby increasing circulating blood volume and blood pressure. Chronic inflammatory conditions can lead to sustained increases in blood pressure by affecting blood vessels and the renal system. Elevated levels of ROS induced by COX upregulation are also associated with processes that can contribute to arterial hypertension through vascular damage and alterations in renal functioning. Hydroxyl radical (HO•) scavengers, including *E. parviflorum*, as noted by Kumagai et al. [19] and Feng et al. [20] inhibit COX regulation and subsequently reduce inflammation.

Early research by Okuda et al. [21] on the antioxidant activity of new classes of tannins showed that the antioxidant properties of hydrolyzable tannins (ellagitannins) were generally higher than those of α-tocopherol and ascorbic acid. Ascorbic acid acts as a strong antioxidant, neutralizing free radicals responsible for damage to the body’s cells and tissues, thereby providing protection against oxidative stress-related damage. Hydrolyzable tannins (ellagitannins) have strong free radical scavenging abilities, which can inhibit the free radical chain reaction of other compounds, through their self-oxidation, thereby preventing the oxidation of lipids, proteins, or DNA, which can significantly impact the development of hypertension [22,23,24,25].

Free radicals can damage the endothelium, leading to vascular dysfunction by reducing NO production and increasing peroxynitrite (ONOO^−^) formation, disrupting the regulation of vascular tone. Reduced NO levels can lead to increased vascular resistance, decreased vasodilation, and elevated blood pressure. Furthermore, tissue damage by free radicals can lead to chronic inflammation in the vascular wall, which in turn increases the production of pro-inflammatory cytokines and chemokines that can affect vascular contraction and increase blood pressure. Damage to DNA and proteins in the endothelium can lead to endothelial dysfunction. Lipid peroxidation leads to the formation of toxic metabolites and damage to cell membranes [19].

Oenothein B inhibits the release of hyaluronidase and myeloperoxidase from stimulated neutrophils, like the anti-inflammatory drug indomethacin [7]. Myeloperoxidase, in the presence of iron ions and hydrogen peroxide (H_2_O_2_), generates oxygen radicals.

Kiss et al. (2012) reported that oenothein B (from *Oenothera paradoxa* extract) can also inhibit lipoxygenase, hyaluronidase, and ROS production from human neutrophils, demonstrating anti-inflammatory activity [26]. On the other hand, high concentrations of oenothein B, according to Sakagami et al., can have a pro-oxidative effect by inducing elevated levels of ROS against human oral tumor cell lines [18]. LOX is an enzyme responsible for the oxidation of fatty acids, leading to the production of leukotrienes. Leukotrienes are lipid mediators of inflammation that can affect vascular tone by inducing smooth muscle contraction and reducing blood flow, contributing to increased blood pressure. Hyaluronidase is an enzyme responsible for the breakdown of hyaluronic acid, which is an important component of the extracellular matrix. Excessive hyaluronidase concentrations may lead to reduced hyaluronic acid levels and affect the permeability and function of the vascular wall as well as the elasticity of blood vessels. On the other hand, hyaluronidase inhibits the release of myeloperoxidase from neutrophils [7]. Therefore, the balance between the synthesis and secretion of this acid is crucial and regulation depends on the concentration of oenothein B [4,26,27].

In another study, the authors evaluated the immunomodulatory properties of tannins by analyzing their effects on human dendritic cells (DCs). The study included measurements of alterations in cytokine production, cell differentiation, and cell viability. Significant dose-dependent changes were observed, including the downregulation of the cell-surface markers CD1a and CD83, the induction of apoptosis in the absence of caspase-3/7, -8, or -9 activation, and the suppression of pro-inflammatory cytokine production—particularly IL-1β and IL-6. These observations may partly account for the traditional use of oenothein B-rich herbal remedies in managing various inflammatory disorders, including rheumatoid arthritis, inflammatory bowel disease, and celiac disease. Moreover, studies conducted by Yoshimura and Okuyama demonstrated that oenothein B mitigates neuroinflammation in the brain as a response to systemic inflammatory stimuli. When oenothein B was administered orally, it acted by enhancing neuronal signaling pathways by (i) suppressing LPS-induced abnormal behavior in open field; (ii) suppressing LPS-induced microglial activation in the hippocampus and striatum; (iii) suppressing LPS-induced COX-2 production in the hippocampus and striatum of mice [28,29,30].

### 3.3. The Impact of Oenothein B on the Liver and the Lipid Profile

High levels of cholesterol in the blood, especially low-density lipoprotein (LDL), have a key role in atherosclerotic plaque development. Elevated LDL cholesterol damages the endothelium of blood vessels, which is the first step of atherosclerosis development [31,32]. Damaged endothelium allows LDL to penetrate the arterial wall, where it undergoes oxidation, triggering an inflammatory response. Oxidized LDL (oxLDL) is recognized by macrophages, which migrate into the arterial wall and ingest oxLDL. These macrophages transform into foam cells filled with lipids, leading to the formation of initial atherosclerotic lesions. Thus, high LDL cholesterol is a major risk factor for atherosclerosis and related cardiovascular diseases [33].

Recently, a plethora of studies focused on liver function in response to either supplementation or direct exposure to different plant extracts and isolated compounds including hydrolyzable tannins. One study demonstrated that strawberry extract (rich in hydrolyzable tannins) added to a high-fat diet reduced oxidative stress, improved lipid profile disturbances, and mitigated inflammatory responses in rats. This manifested as decreased triglycerides and total cholesterol. Moreover, body weight gain, hepatic fat, oxidized glutathione, and thiobarbituric acid-reactive substance concentrations also decreased [34]. In another study conducted by the same research team, ellagitannin-rich extract obtained from strawberries induced beneficial changes in the hepatic fat content, reduced and oxidized glutathione (GSH and GSSG) concentrations, the GSH/GSSG ratio, the modified blood plasma antioxidant status (FRAP, ACL), HDL-cholesterol, and the atherogenic coefficient values in rats fed with a high-fat diet [24].

Hydrolyzable tannin (oenothein B) treatment has protective effects and supports liver function. Oenothein B ameliorates hepatic injury in mice with alcoholic liver disease by improving oxidative stress and inflammation and modulating the gut microbiota. Xu et al. demonstrated that oenothein B treatment mitigated alcohol-induced liver damage, as indicated by lower levels of inflammatory biomarkers, aminotransferases and restored gut microbiota dysbiosis [35]. Oenothein B mitigated pathological damage by reducing lipid droplet accumulation, lipid vacuolation, inflammation, and fibrosis. The authors concluded that supplementation with oenothein B is involved in preserving hepatocyte integrity and modulating lipid metabolism in these cells. Oenothein B increased the expression of NQO1 genes, Hemoxygenase-1 (HO-1), Nuclear factor erythroid 2-related factor 2 (Nrf2), and Kelch-like ECH-associated protein 1 (Keap1) levels. The Keap1/Nrf2 signaling pathway is a crucial component of the liver’s antioxidative system with protective effects. Under normal conditions, Keap1 binds to Nrf2, promoting its ubiquitination and degradation in the cytoplasm, maintaining low Nrf2 levels. During inflammation and oxidative stress, Nrf2 dissociates from Keap1 and translocates into the nucleus, promoting the expression of target genes such as HO-1, NQO1, SOD, CAT, and GSH, which reduce oxidative stress-induced damage [35]. Oenothein B increases the levels of antioxidant enzymes (SOD, CAT, GSH) and decreases oxidative stress markers (MDA, CYP2E1) [35]. This indicates that oenothein B effectively reduces oxidative stress in the liver. In vivo studies have demonstrated that in HepG2 cells, levels of SOD, CAT, and GSH were lower, while MDA and ROS levels were higher, confirming the impact of oenothein B on reducing oxidative stress.

Oenothein B plays an important role in reducing atherosclerotic plaque formation through several mechanisms related to regulating inflammation, oxidative stress, and gut microbiota. Oxidative stress and inflammation play a key role in the pathogenesis of alcoholic liver disease (ALD). Acute alcohol consumption increases gut permeability, allowing lipopolysaccharides (LPS) to enter the liver, where they activate toll-like receptor 4 (TLR4) on Kupffer cells. TLR4 activation leads to increased expression of inflammatory genes such as NF-κB, Myd88, and CD14, resulting in the production of inflammatory cytokines (IL-1β, IL-6, TNF-α), which cause liver inflammation and damage. Oenothein B significantly reduces TLR4 expression and downstream inflammatory mediators, leading to decreased levels of inflammatory cytokines in the liver. This anti-inflammatory effect has also been confirmed in vitro, where oenothein B reduced ethanol-induced NO levels in HepG2 cells. Oenothein B limits the nuclear translocation of p65, reducing NF-κB binding activity and inhibiting TLR/NF-κB pathway-dependent NO synthesis [35]. Thus, oenothein B alleviates oxidative stress and inflammation, which are crucial factors in atherosclerotic plaque development. Alcohol abuse alters gut microbiota composition, leading to disturbances of the gut–liver axis. These changes can influence the development of inflammation and oxidative stress in the body. Supplementation with oenothein B restores the gut microbiota balance by increasing the concentration of short-chain fatty acid (SCFA) producers such as a family of bacteria within *Muribaculaceae* and *Erysipelotrichaceae*. SCFAs are gut microbiota metabolites that activate the Nrf2 signaling pathway in the liver, enhancing the antioxidative system. Oenothein B reduces the abundance of Gram-negative bacteria (*Akkermansia*), whose lipopolysaccharide (LPS) activates the TLR4/NF-κB signaling pathway in the liver, triggering an inflammatory response. Reducing the number of these bacteria decreases overall inflammation in the body [35].

In summary, oenothein B plays a multifaceted role in reducing atherosclerotic plaque by regulating oxidative stress, reducing inflammation and restoring gut microbiota balance. Through modulation of these processes, oenothein B may contribute to improving vascular health and reducing the risk of atherosclerosis.

### 3.4. Epilobium and Its Active Compounds Induce Apoptosis of Cancer Cells

Aqueous extracts from *Epilobium parviflorum* Schreb., *E. angustifolium* L., and *E. hirsutum* L. herbs induce apoptosis in human hormone-dependent prostate cancer cells through activation of the mitochondrial pathway and caspase-3 activation [5]. In this process, disruption of the mitochondrial membrane potential occurs, triggering the release of cytochrome c from the mitochondria into the cytosol. In the cytoplasm, cytochrome c binds with Apaf-1 (apoptosis protease-activating factor-1) and pro-caspase-9, forming a complex called the apoptosome. Upon formation of the apoptosome, pro-caspase-9 is activated and converted into caspase-9, which in turn activates other caspases, such as caspases-3, -6, and -7, through the proteolytic conversion of proenzymes into their active forms. The activation of caspases leads to the fragmentation of cellular proteins, DNA degradation [18], changes in cytoskeletal structure, and the formation of apoptotic vesicles, resulting in controlled cell breakdown without inducing inflammation [36].

Extracts of various species of Epilobium inhibit the proliferation of human prostatic epithelial cells in vitro by affecting the progression of the cell cycle [37]. The active compounds, hydrolyzable tannins (ellagitannins), inhibit cancer cell growth by suppressing proliferation and inducing apoptotic cell death [18]. They exhibit cytotoxicity against cancer cell lines by disrupting the balance between proliferation and apoptosis. In this process, induction of apoptotic cell death occurs through cleavage of cytokeratin 18 and DNA fragmentation by activated caspases [18]. Furthermore, oenothein B is a potent inhibitor of aromatase and 5α-reductase, enzymes implicated in the pathogenesis of benign prostatic hyperplasia [38]. This prevents the development of inflammation and the disturbance of cell division balance within the prostate gland. Oenothein B induces neutral endopeptidase in PC-3 prostate cancer cells, which inactivates growth-stimulating neuropeptides. Oenothein B is responsible for inhibiting poly(ADP-ribose) polymerase on specific chromosomal proteins in eukaryotic cells, mainly through poly(ADP-ribose) glycohydrolase. This process is a significant factor in regulating gene activation, DNA replication, transcription, and cell death. Hydrolyzable tannins (ellagitannins) exert an antimutagenic activity toward the direct mutagen medication mitomycin C [39]. In another study, antigenotoxic and anticytotoxic effects against DNA damage induced by cyclophosphamide were studied [40]. The authors concluded that oenothein B protects DNA from CPA-induced damaging factors and induces DNA repair after damage [40]. Additionally, Miyamoto et al. concluded that hydrolyzable tannins (ellagitannins) with a marked antitumor activity should possess a dimeric structure with several galloyl groups on the glucose core [41]. Later authors demonstrated that oenothein B has anticancer activity through host-mediated induction of cytotoxic peritoneal exudate cells, via the activation of macrophages and secreting interleukin 1β [42]. Agrimoniin, an antitumor tannin of *Agrimonia pilosa* Ledeb. (Rosaceae), like oenothein B induces interleukin-1 [43,44]. Evening primrose (*Oenothera biennis* L, Onagraceae) root extract and its active compound, oenothein B, target the PD-1/PD-L1 blockade in experiments conducted in vivo utilizing a colorectal cancer mouse model [45].

Stolarczyk et al. [5] demonstrated that macrocyclic hydrolyzable tannins (ellagitannins), including oenothein B, may exhibit prooxidant activity in prostate cancer cells. Oenothein B induces an increase in ROS levels [5,18]; this, in turn, could trigger the initiation of intrinsic apoptosis through mitogen-activated protein kinase and/or phosphatidylinositol 3-kinase pathways. In response to increased ROS levels, cells can initiate defensive and signaling mechanisms that may beneficially affect their ability to adapt to changing environmental conditions. However, excessive ROS and its chronic maintenance can lead to oxidative stress, where the balance between ROS production and the body’s antioxidant mechanisms is disrupted in favor of ROS. Oxidative stress is associated with accelerated aging processes and the development of many chronic diseases, including hypertension. Therefore, understanding the impact of the concentration and duration of supplementation with extracts from Epilobium herbs requires further research.

Oenothein B participates in the release of TNF-α, IL-2, and IL-1β, and stimulates peripheral blood mononuclear cells (PBMCs) in a dose-dependent manner [46]. It also activates T lymphocytes through IL-1β, which are responsible for eliciting a host-mediated anticancer response rather than direct cytotoxic effects on cancer cells [44]. Oenothein B increases the expression of BAX, BAK, and BAD, p53, cytochrome c (cytoplasmic), and PARP. It also activates caspase-3 and 9, and decreases the expression of BCL-2, FADD, TRADD, TNFRSF10A, and TNFRSF10C [46]. In another study, extracts from five Epilobium species (*E. hirsutum* L., *E. parviflorum* Schreb., *E. palustre* L. *E. dodonaei* Vill., and *E. angustifolium* L.) demonstrated strong antioxidant activity as evaluated by DPPH and TEAC assays [47]. Among the tested optimized extracts, *E. angustifolium* aerial parts showed the greatest selectivity in targeting cancerous cells (prostate carcinoma cell lines), followed by an *E. hirsutum* (commonly known as hairy willowherb) aerial parts extract. For antioxidant activity, extracts from *E. hirsutum* leaves and aerial parts exhibited the highest potency in reducing ROS levels. Extracts from the aerial parts of *E. dodonaei* and *E. angustifolium* leaves exhibited the strongest anti-inflammatory effects, as measured by IL-6 and IL-8 levels [47]. *E. hirsutum* L. extract also exhibited antitumor and anti-inflammatory effects in the studied animal models [48].

Epilobium possesses cytotoxic properties, impacting reductions in malignant melanoma (an aggressive type of skin cancer) [49], sarcoma [17], breast cancer (MCF-7) [50], prostate cancer (PC-3 and LNCaP) [38], lung cancer (A549), and colon cancer (HT-29); as well as glioblastoma (1231N1), neuroblastoma (SK-N-SH), human oral cavity epidermis (KB), cervical cancer (HeLa), and liver cancer when tested on these cell lines (Hep-3B), with lower cytotoxicity towards normal cell lines (WISH) [49].

### 3.5. Nuclear Factor (NF-κB) Versus Epilobium Active Compounds

Oenothein B (from *E. angustifolium*) is responsible for the induction of intracellular Ca^2+^ flux, the activation of NF-κB, and the production of ROS and pro-inflammatory cytokines [46]. A low level of ROS is essential for maintaining redox balance and cell proliferation. High levels of ROS can activate PI3K/Akt signaling mainly by inhibiting phosphatases such as PTEN or directly activating oncogenes, including AKT. The PI3K/Akt signaling pathway is a mediator of NF-κB, which plays a significant role in cell proliferation, cell cycle progression, and cell viability in cancer. By increasing ROS levels, oenothein B may prevent cell proliferation and stimulate the cell death of lung cancer, likely through the PI3K/Akt/NF-κB signaling pathway. This is the opposite effect to ROS inhibitors like N-acetyl-L-cysteine and the PI3K agonist (insulin-like growth factor 1, IGF-1) [51]. It has also been noted that oenothein B can induce keratinocyte growth and stimulate neutrophil influx.

It has been proven that oenothein B dose-dependently reduces NO production, inducible nitric oxide synthase (iNOS), and iNOS protein levels without inhibiting iNOS enzymatic activity using murine macrophages [16,52]. This action is NF-κB dependent, but independent of the interferon (IFN)-γ/JAK-STAT pathway. As is known, improper or excessive NO production by iNOS is closely linked to numerous inflammatory diseases, making oenothein B a promising lead in developing therapeutic agents as effective inhibitors of NO production.

### 3.6. Lymphocytes Cells and Interferons (IFNs) Versus Epilobium Active Compounds

Ramstead et al. reported that oenothein B stimulated innate γδ T cells, αβ T cells, and NK cells, leading to increased expression of CD25 and/or CD69. This pathway increases IFN-γ production by NK cells and T lymphocytes [53]. In another study, aging influenced the response of T cells to stimulation by oenothein B [54]. Studies by Caillon et al. determined that a small percentage of γδ T lymphocytes in circulation contribute to hypertension mediated by angiotensin II [55]. Gene expression microarray analysis of whole blood in patients with or without coronary artery disease revealed a positive correlation between the frequency of γδ T cells in peripheral blood and systolic blood pressure. Yoshimura et al. noted that oenothein B dose-dependently reduces IFN-γ levels, suggesting its involvement in regulating systolic pressure through changes in IFN-γ levels [28].

### 3.7. Dendritic Cells Versus Epilobium Active Compounds

Yoshimura et al. also highlighted the immunomodulatory properties of oenothein B on human dendritic cells (DCs), which reside in tissues exposed to external factors, including the lungs, skin, gastrointestinal tract, nasal mucosa, and intestines [28]. These cells are essential in initiating the immune response through their function as antigen-presenting cells. Immature dendritic cells (DCs) possess strong phagocytic capabilities, and upon antigen uptake and maturation, mature DCs migrate to the lymph nodes where they present antigens to naive T cells. Macrophages, dendritic cells (DCs), and B cells participate in antigen presentation via MHC class II molecules. Another important function associated with DCs is their constitutive role in initiating inflammation related to certain autoimmune diseases, including celiac disease, inflammatory bowel diseases, and rheumatoid arthritis. Oenothein B downregulates CD1a and CD83 cell surface molecules and the cytokines IL- 1β, IL-6, IL-12, IL-17, IFN-γ, and MIP-1β, dose-dependently inhibiting inflammatory processes. Although oenothein B has been noted to accelerate inflammatory cytokine release from monocytes, significant suppression of IL-6 production below detectable levels and the downregulation of IL-1β may thus induce the anti-inflammatory effects of tannins via DCs. The study found that tannins have an anti-inflammatory effect at the peripheral level by reducing the concentration of cytokines, disrupting the function of dendritic cells and stimulating their apoptosis.

Oenothein B suppresses cell surface molecules, delaying antigen presentation, lowering cytokine production, and inducing their apoptosis. The activation of caspases-3/7, 8, and 9 suggests a caspase-dependent mechanism of this apoptosis [10] [20].

### 3.8. Brain Inflammation Versus Epilobium Active Compounds

Okuyama et al. suggested that oenothein B may reduce neuroinflammation during systemic inflammation [29,30]. Oenothein B itself barely crosses the blood–brain barrier (BBB), but metabolites produced by gut microflora can cross the BBB and act directly in the brain as anti-inflammatory agents. Hydrolyzable tannins (ellagitannins) are converted in the intestine to ellagic acid (2,3,7,8- tetrahydroxy-benzopyrano [5,4,3-cde] benzopyran-5-10-dione), which is then converted into metabolites such as urolithins (i.e., urolithin A, 3,8- dihydroxyurolithin, and urolithin B, 3-hydroxyurolithin) by gut bacteria [56]. According to recent studies, ellagic acid has neuroprotective functions in the brain, preventing cognitive deficits, hippocampal deficits, and amnesia. Urolithin A performs neuroprotective functions, protecting against ischemic neuronal damage by enhancing autophagy.

### 3.9. cAMP-Responsive Transcription Factor (CREB) Versus Epilobium Active Compounds

Oenothein B activates CREB by phosphorylating the serine residue via various kinases, such as Ca^2+^/calmodulin-dependent protein kinase, Akt/protein kinase B, protein kinase A, and glycogen synthase kinase-3 [29,30,55]. Garat et al. reported that CREB depletion in smooth muscle cells contributes to medial thickening, adventitial fibrosis, and pulmonary hypertension. CREB deficiency led to structural and hemodynamic changes characteristic of pulmonary hypertension (PH) in vivo. Rats with reduced CREB levels showed serum-independent proliferation and hypertrophy in vitro, and they secreted soluble factors that stimulated proliferation and extracellular matrix protein expression by adventitial fibroblasts. This indicates that CREB plays a crucial role in regulating the pathological transformation of arterial smooth muscle cells (SMCs) from a homeostatic, resting form to a proliferative, synthetic form, driving arterial remodeling and contributing to PH development [57].

### 3.10. Antibacterial and Anti-Inflammatory Properties of Epilobium and Epilobium Active Compounds

Epilobium extracts possess antiseptic properties effective against the fungi *Candida albicans*, *Microsporum canis*, and *Trichophyton tonsurans*, and the dermatophytes *Arthroderma* spp. (*E. angustifolium*) [58]. They are beneficial for skin ulcers, swelling [59], and wound healing (*E. angustifolium*) [4].

Moreover, several reports have characterized the beneficial effects of Epilobium hydrolyzable tannins (ellagitannins) towards a variety of bacteria: *Escherichia coli* [1,58], *Staphylococcus aureus* [58,60], *Pseudomonas aeruginosa* [58,59], *Bacillus cereus*, *Micrococcus luteus*, *Klebsiella pneumoniae,* and *Acinetobacter baumannii* [58], as well as the protozoa *Leishmania donovani* [61], and the fungi *Paracoccidioides brasiliensis* [62]. Particularly noteworthy is the synergistic action of polyphenols with antibiotics against antibiotic-resistant bacteria. Methicillin-resistant *Staphylococcus aureus* (MRSA) has shown sensitivity to β-lactam antibiotics combined with oenothein B [60].

## 4. Conclusions

*E. parviflorum* offers a wide range of health benefits, primarily due to its rich content of bioactive compounds like flavonoids, phenolic acids, and hydrolyzable tannins (oenothein B). Its applications in traditional and modern medicine highlight its potential in treating prostate disorders, reducing inflammation, combating oxidative stress, and offering anticancer and antimicrobial effects. The presence of oenothein B in *E. parviflorum* may influence the production and storage of NO, which in turn promotes vasodilation and blood pressure regulation. Moreover, extracts from Epilobium alleviate the effects of excess ROS and inflammation, thereby protecting against oxidative stress and related disorders. The limited number of experimental studies regarding the effects of *E. parviflorum* on the vascular system justifies the need for further scientific research in this field. Therefore, it seems reasonable to pursue additional studies on *E. parviflorum* to fully understand its therapeutic potential, useful for the development of preparations benefiting vascular health.

## Figures and Tables

**Table 1:** Vascular research results.

Study	Plant	Material	Model (Ex Vivo/In Vitro)	Intervention	Duration	Parameters Measured	Effect	Mechanism
Isla et al., 2024 [10]	*Cuphea carthagenensis* (Jacq.) J. F. Macbr (Lythraceae)	Aqueous extract (AE)	Supplementation to rats	0.5 and 1.0 g/kg/day	1 week	Systolic blood pressure (non-invasive tail-cuff method)	Hypotensive effect	Unknown; ellagitannin-independent due to the low oral bioavailability of hydrolyzable tannins (ellagitannins)
		Oenothein B, woodfordin C, and eucalbanin B isolated from AE	Ex vivo—rat aortic rings pre-contracted with the vasoconstrictor noradrenaline	20–180 µM		Vasorelaxation	Concentration-related vasorelaxation	Endothelium-dependent via activation of nitic oxide (NO) synthesis and/or NO release from endothelial cells without alteration of Ca^2+^ influx in vascular smooth muscle preparations

## Data Availability

Data is contained within the article.
